# Kelulut Honey Ameliorates Oestrus Cycle, Hormonal Profiles, and Oxidative Stress in Letrozole-Induced Polycystic Ovary Syndrome Rats

**DOI:** 10.3390/antiox11101879

**Published:** 2022-09-22

**Authors:** Datu Agasi Mohd Kamal, Siti Fatimah Ibrahim, Azizah Ugusman, Mohd Helmy Mokhtar

**Affiliations:** 1 Department of Physiology, Faculty of Medicine, Universiti Kebangsaan Malaysia, Kuala Lumpur 56000, Malaysia; 2 Department of Biomedical Sciences, Faculty of Medicine and Health Sciences, University Malaysia Sabah, Kota Kinabalu 88400, Malaysia

**Keywords:** kelulut honey, antioxidative, honey physicochemical properties, PCOS

## Abstract

Kelulut honey (KH) has been proven to have excellent antioxidative and anti-inflammatory properties with unique physicochemical characteristics. Therefore, we investigated the isolated and combined effects of KH, metformin, or clomiphene in alleviating oxidative stress and reproductive and metabolic abnormalities in polycystic ovary syndrome (PCOS). Female Sprague-Dawley (SD) rats were given 1 mg/kg/day of letrozole for 21 days to induce PCOS. PCOS rats were then divided into six treatment groups: untreated, metformin (500 mg/kg/day), clomiphene (2 mg/kg/day), KH (1 g/kg/day), combined KH (1 g/kg/day) and metformin (500 mg/kg/day), and combined KH (1 g/kg/day) and clomiphene (2 mg/kg/day). All treatments were administered orally for 35 days. The physicochemical characteristics of KH were assessed through hydroxymethylfurfural, free acidity, diastase number, moisture content, sugar profile, metals, and mineral compounds. Additionally, we determined the semivolatile organic compounds present in KH through gas chromatography-mass spectrometry (GC/MS) analysis. KH and its combination with metformin or clomiphene were shown to improve the oestrus cycle, hormonal profile, and oxidative stress in PCOS rats. However, KH did not reduce the fasting blood glucose, insulin, and body weight gain in PCOS rats. These findings may provide a basis for future studies to discover the potential use of KH as a complementary treatment for women with PCOS.

## 1. Introduction

Kelulut honey (KH) is multi-floral stingless bee honey from *Trigona* spp. Over 500 species of stingless bees and 64 distinct genera have been found worldwide [1]. Stingless bees habituate the warm and humid forests and are often found in tropical countries. KH is native to Southeast Asia and can be found in Malaysia, Indonesia, and Thailand. This honey has been used traditionally as a remedy for anti-ageing and improving the immune system in these countries. The physicochemical properties of KH are unique. It differs from the Apis honeybees; hence, its quality should not be assessed according to the standards of the Apis honeybees, such as Codex Alimentarius and the International Honey Commission (2009). In addition, numerous studies have shown that stingless bee honey does not meet the quality standards for Apis bee honey [2], and the International Standard for stingless bee honey has still not been established. However, in 2017, Malaysia published a quality standard for Malaysian stingless bees as quality control for marketed stingless bee honey [3]. 

KH has demonstrated beneficial health effects as evidenced by numerous studies. For example, KH is reported to have high antibacterial potency [4], chemopreventive properties in the colorectal cancer rat model [5], and anti-inflammatory properties by protecting against lipopolysaccharide (LPS)-induced chronic subclinical systemic inflammation in rats [6]. KH also prevents keloid scar formation by attenuating TGFβ-induced epithelial to mesenchymal transition in primary human keratinocytes and improves ethanol-induced gastric ulcers in rats [7]. Additionally, KH shows anti-obesity properties by improving lipid and cholesterol profiles [8,9]. 

The most prominent property of KH is its antioxidative actions. In many settings, KH has demonstrated excellent antioxidative activities. A previous study reported that treatment with KH improved the oxidative damage in streptozotocin-induced diabetic rats by increasing superoxide dismutase (SOD) activity and glutathione (GSH) levels and decreasing the protein carbonyl (PC) and malondialdehyde (MDA) levels in the sperm and testis of the diabetic rats [10]. Another study showed that KH could ameliorate glucocorticoid-induced osteoporosis by reducing lipid peroxidation [11]. In LPS-induced chronic subclinical systemic inflammation in rats, KH treatment significantly improved the levels of oxidative stress markers such as MDA, 8-hydroxy-2′-deoxyguanosine (8-OHdG), GSH, glutathione peroxidase (GPx), and glutathione S-transferase (GST) [6]. In fact, several studies have found that KH possesses a higher antioxidant content than common bee honey such as Acacia and Tualang honey [12,13,14]. Trehalulose, a highly active antioxidant, is one of the discovered content in KH that is responsible for its antioxidative actions [15]. Taken together, KH has been shown to have potential therapeutic benefits for a variety of diseases related to oxidative stress. 

Polycystic ovary syndrome (PCOS) is a complex disorder that affects the reproductive, endocrine, metabolic, and psychological systems [16]. The anomalies of ovarian steroidogenesis, insulin resistance, hyperinsulinism, abnormalities of gonadotrophin production, and follicular arrest are some of the aetiologies of PCOS [17,18]. Additionally, it has been demonstrated that low-grade inflammation and oxidative stress play a role in the aetiology of PCOS [19,20,21]. Symptoms of PCOS include hyperandrogenism, anovulation, infertility, obesity, menstrual cycle irregularities, dyslipidaemia, and hirsutism [22]. Treatment for patients with PCOS typically focuses on the symptoms, as there is yet no definitive treatment for PCOS [23]. 

Medications used to treat PCOS include clomiphene citrate and metformin [24]. However, these medications are linked to a number of side effects, such as diarrhoea, vaginal and uterine bleeding, breast discomfort, hot flashes, and abdominal pain [25]. Therefore, discovering a natural supplement that could be used as a complementary treatment for PCOS with minimal side effects is of great interest. Previously, inositol, a naturally occurring substance found primarily in fruits, grains, and beans, was shown to be effective in treating metabolic and reproductive disorders in PCOS women [26,27]. This finding strengthens the potential use of nutraceuticals in treating typical symptoms of PCOS and the need for more research to be conducted in this field. Recently, we found that KH could improve the regulation of the oestrus cycle and ovarian histomorphological alterations in rats with letrozole-induced PCOS [28]. In view of these promising effects, the current study aimed to focus on the antioxidative effect of KH on letrozole-induced PCOS rats. Additionally, we demonstrated the physicochemical properties of KH, including the semivolatile component, to verify the quality and discover the potential beneficial compound in KH.

## 2. Materials and Methods

### 2.1. Honey Sample

Kelulut honey (KH) was gathered in Negeri Sembilan, Malaysia, by an experienced local beekeeper. The nearby herbal plant provides nectar for the bees to collect. The KH was kept raw at 4 °C in amber bottles away from heat sources and sunlight until further analysis.

### 2.2. Physicochemical Profiling of KH 

#### 2.2.1. Moisture Content

The Petri dish was weighed empty before adding 1 g of KH. The uncovered dish was then placed in an oven at 60 °C for two hours. Subsequently, the dish was transferred into a desiccator to cool it before reweighting. The heating and weighing steps continued for another two hours in the oven until a weight change of less than 2 mg was achieved. The moisture content was determined using the following formula:

(1) $$\mathrm{Moisture}\;\mathrm{content}:\;\frac{W_{2}-W_{1}}{W}\times 100$$

Moisture content: $\frac{W_{2}-W_{1}}{W}\times 100$

*W*_2_ = Weight of empty dish + sample (g)

*W*_1_ = Weight of dried dish + dried sample (g)

*W* = Weight of the sample (g)

#### 2.2.2. Ash 

The ash content was calculated by weighing 5 g of KH samples in a platinum dish and heating them to a consistent weight in a laboratory furnace at 600 °C following the AOAC Method 920.181 [29]. Ash content was measured in triplicate. Total ash content was measured in triplicate and expressed as the percentage of residue left after dry oxidation by weight (g/100 g honey), which was calculated using the following equation:

Ash(%) = [(m_1_ − m_2_)/m_0_] × 100 (2)

Note that m_0_ is the mass of the KH taken, m_1_ is the mass of the dish plus ash, and m_2_ is the mass of the platinum dish before it was calcined.

#### 2.2.3. Free Acidity

The titrimetric method was used to determine free acidity following the AOAC Official Method 962.19 [29]. In a 250 mL beaker, 10 g of KH sample was dissolved in 75 mL CO_2_-free water. The solution was swirled with a magnetic stirrer while the electrode of the pH meter was submerged and titrated to pH 8.5 by adding 0.05 M NaOH solution. 

#### 2.2.4. Diastase

Diastase activity was calculated according to the AOAC Official Method 958.09 [29]. A buffered mixture of soluble starch and KH was incubated in a thermostatic bath at 40 °C. Subsequently, 1 mL aliquot was taken at 5 min intervals, and a Perkin Elmer Luminescence Spectrophotometer was used to measure the sample’s absorption at 660 nm (Norwalk, CT, USA). The diastase number was determined using the same length of time it took for the absorbance to reach 0.235. The results were reported in Schade units per gram of honey as the volume (mL) of 1% starch hydrolysed by an enzyme in 1 g of honey in 1 h.

#### 2.2.5. Minerals and Metals

Determination of minerals and heavy metals was performed using inductively coupled plasma–optical emission spectrophotometry (ICP-OES) described by Aghamirlou et al. [30]. Briefly, samples were weighed at approximately 1 g. The sample was then mixed with 10 mL of HNO_3_ and heated for 1 h and 30 min at 95 °C in a microwave. After cooling to room temperature, 50 mL of distilled water was added, and the sample filtrate was analysed by ICP-OES (Agilent 7500ce, Agilent Technologies Inc. Palo Alto, CA, USA). Table 1 shows the ICP-OES operating conditions.

#### 2.2.6. Determination of Hydroxymethylfurfural (HMF)

The method of [31] was used to determine the HMF content. A 50 mL volumetric flask containing 0.5 mL of Carrez solution I and 0.5 mL of Carrez solution II was filled with precisely 5 g of KH dissolved in 25 mL of water. Water was added to the flask to make it 50 mL larger, and the resultant solution was then filtered. After discarding the first 10 mL of filtrate, two aliquots of 5 mL each were added to the test tubes. The reference tube received 5 mL of a 0.2% sodium bisulphite solution, whereas the sample tube received 5 mL of pure water. A UV-visible spectrometer was used to measure the solution’s absorbance at two wavelengths, 284 and 336 nm. The equation below was used to determine the HMF content:

(3) $$\mathrm{HMF}\;(\mathrm{mg}/100\;g)=\left(A_{284}-A_{336\;}\right)\;\times \;14.97\;\times \;5$$

Note that 14.97 was the factor, and 5 was the mass of the sample. *A*_284_ was the absorbance at 284 nm, and *A*_336_ was the absorbance at 336 nm.

#### 2.2.7. Sugar Profiling

The high-performance ion chromatography (HPIC)-based approach, as described in [32], was used to profile the sugar levels in the KH samples. The amount of 200 mg of KH was dissolved in a few millilitres of water. This solution was transferred quantitatively into a volumetric flask, filled to the 100 mL mark with water, thoroughly mixed, and then filtered through a 0.02 m nylon membrane filter (Whatman). The sample was then injected into the Thermo Scientific™ Dionex™ ICS-5000+ machine and detected by an electrochemical detector. HPIC-grade solvents were applied to a Dionex CarboPac PA20, Analytical (3 × 150 mm) column from Thermo Fisher Scientific in Waltham, MA, USA. 

#### 2.2.8. Semivolatile Organic Compound (SVOC) Determination

Gas chromatography-mass spectrometry (GC/MS) was used for SVOC determination on the KH sample based on modifications to the U.S. E.P.A. 8270 protocol [32]. The KH samples were extracted with 99.8% dichloromethane (GC grade, Merck, Darmstadt, Germany) before concentrating on the minimum injection volume. The sample was injected into the splitless inlet of an Agilent 7890B, 5977B MSD GC-MS system (Agilent Technologies, Santa Clara, CA, USA). The carrier gas, helium, flowed at a 1.0 mL/min rate. The peaks collected were compared with those in the NIST collection to identify the SVOCs (Gaithersburg, MD, USA). Table 2 lists the GC and MS running circumstances.

### 2.3. Animal Preparation

The ethical review and approval of the study protocol were granted by the National University of Malaysia Animal Ethics Committee (Ethical Approval Code FISIO/FP/2020/MOHD HELMY/14-MAY/1104-JUNE-2020-MAY-2023). Female Sprague–Dawley (SD) rats weighing 120–150 g and exhibiting at least two consecutive regular oestrus cycles were used in this study. The experimental animals were supplied by Laboratory Animal Research Unit (LARU), Faculty of Medicine, Universiti Kebangsaan Malaysia. Rats were kept in individual cages, allowed to acclimate for a week, kept in an air-conditioned room at 24 ± 2 °C with a 12 h light/12 h dark cycle, and provided with regular food pellets and water ad libitum. Animals were weighed twice a week, and the oestrus phase was determined daily by observing vaginal smears under an Olympus BX40 light microscope (Olympus Corporation, Tokyo, Japan).

#### Animal Treatment 

**Figure 1:**
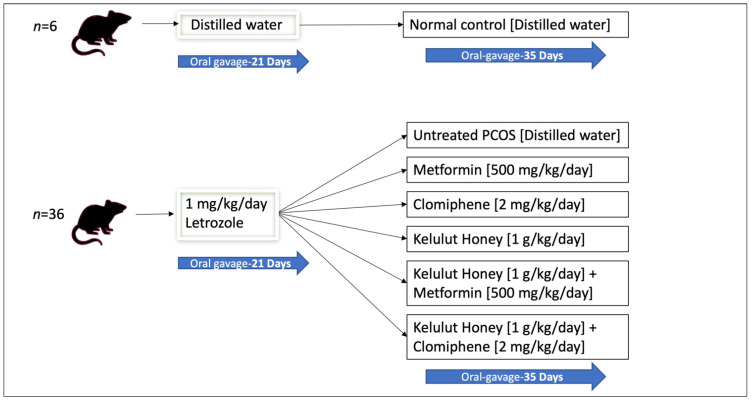
A schematic diagram of the animal grouping and treatments.

The rats were divided into two main groups (Figure 1). The first group (normal control group, *n* = 6) received distilled water throughout the study (56 days). The second group (*n* = 36) was administered with 1 mg/kg/day of letrozole orally, once daily for 21 days, to induce PCOS as reported in a previous study [33]. As validated in our previous study, PCOS was induced in all rats administered with 1 mg/kg/day of letrozole for 21 days, determined by examining the dioestrous days, blood glucose levels, and ovarian histology [28]. 

The PCOS rats (*n* = 36) were then randomly distributed into six experimental groups (*n* = 6 per group): untreated PCOS rats that received distilled water, treatment with metformin (500 mg/kg/day), treatment with clomiphene (2 mg/kg/day), treatment with KH (1 g/kg/day), combined treatment with KH (1 g/kg/day) and metformin (500 mg/kg/day), and combined treatment with KH (1 g/kg/day) and clomiphene (2 mg/kg/day). All treatments were administered orally for 35 days. The doses of metformin (500 mg/kg/day) and clomiphene (2 mg/kg/day) were based on the study by Ndeingang et al. [34]. KH dose (1 g/kg/day) and treatment duration were determined from our pilot study [28]. Metformin is used for insulin resistance in women with PCOS, whereas clomiphene is used to induce ovulation [24]. We designed the groups to receive metformin and clomiphene or a combination of these drugs with KH to assess the effect of KH treatment and to investigate any synergistic effect. All animals were euthanised by ketamine-xylazine overdose (0.3 mL/100 g body weight) at the end of the 35 days [35]. 

### 2.4. Determination of Oestrous Cycle

Every day at 9:00 am, all the rats had vaginal smears taken using cotton buds dipped in 0.9% saline. The vaginal fluid was then collected by rolling a cotton bud onto a glass slide. Oestrous cycles were tracked until the end of the study. The cells were stained with methylene blue and viewed under a light microscope. The smears were categorised as one of the four oestrous cycle phases previously described [36]. A smear of the proestrous phase has a significant number of rounded and nucleated epithelial cells. The oestrous phase, in contrast, is characterised by smears that primarily contain irregular cornified cells without a nucleus. Leukocytes were mainly found in the dioestrous phase and were tiny, rounded cells without a nucleus. The metoestrous phase is characterised by having the same ratio of leukocytes, cornified cells, and nucleated epithelial cells in a smear [36].

### 2.5. Determination of Fasting Blood Glucose 

This test was performed before the rats were sacrificed. Rats were fasted for eight hours, and a sample of tail blood was used to measure blood glucose using a handheld glucometer (Accucheck performa, Roche Diagnostics, Basel, Switzerland).

### 2.6. Determination of Serum Hormone Levels and Insulin by Enzyme-Linked Immunosorbent Assay (ELISA) Technique

Prior to rat sacrifice, samples of retro-orbital blood were collected in serum separator tubes (BD Vacutainer SSTTM, Becton Dickinson, Franklin Lakes, NJ, USA). Samples were allowed to clot at 4 °C before centrifugation at 3000× *g* for 15 min and stored at −80 °C until hormonal analysis was performed.

The serum hormone levels were analysed using a competitive ELISA for oestradiol, progesterone, and testosterone and a sandwich ELISA for follicle-stimulating hormone (FSH), luteinising hormone (LH), and insulin. All samples were tested in duplicate according to the manufacturer’s guidelines (Elabscience, Houston, TX, USA). In brief, reference wells were prepared by mixing biotinylated antibody solution and reference standards. Biotinylated antibody solution and serum samples were added to the test wells. The plate was then sealed and incubated for 45 min at 37 °C. The plate washing was done with a wash buffer and repeated thrice. Horseradish peroxidase (HRP) conjugate was added to each well, and the plate was incubated for 30 min at 37 °C. Then, the plate washing process was repeated five times. A substrate reagent was then added to each well, and the plate was incubated for 15 min at 37 °C. Finally, a stop solution was added to each well to stop the reaction, and the absorbance of the solution in the wells was immediately measured at 450 nm using a microplate reader. Standard dilutions of known hormone concentrations were used to create the standard curve. The produced standard curve was used to determine the serum hormone concentrations. 

### 2.7. Oxidative Stress Status Evaluation

Ovarian tissue lysates were prepared based on previous methods [37]. The tissues were weighed and powdered in liquid nitrogen using a mortar and pestle. The tissue powder was then mixed with phosphate-buffered saline (PBS; 0.1 M, pH 7.4) in a ratio of 1:9 (*w*/*v*), and centrifuged for 10 min at 4 °C. The protein concentration in the ovarian tissue lysates was measured using the Bradford assay [38].

Levels of catalase (CAT, as U/mg protein), total superoxide dismutase (SOD, as U/mg protein), glutathione peroxidase (GSH, as mg U/mg protein), and malondialdehyde (MDA, as µmol/g protein) in ovarian tissues were determined using calorimetric diagnostic kits (Elabscience, Houston, TX, USA) according to the manufacturer’s guidelines.

### 2.8. Statistical Analysis

The data were reported as mean ± SEM. GraphPad Software (GraphPad Inc., San Diego, CA, USA) was used to determine the differences between the groups using one-way ANOVA and Tukey’s multiple comparison tests. Statistical significance was defined as *p* < 0.05.

## 3. Results

### 3.1. Physicochemical Profile of KH for Quality Determination 

**Figure 2:**
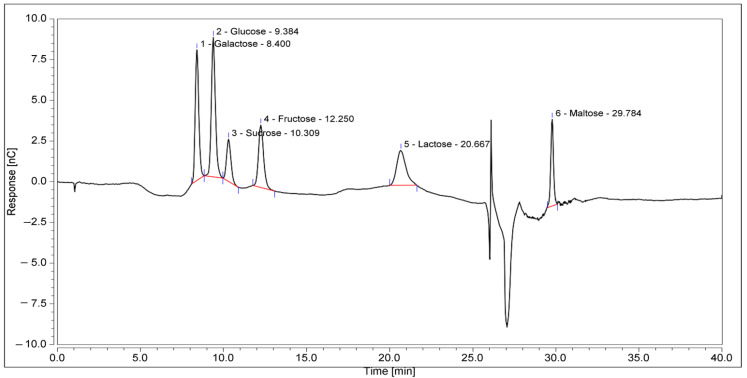
HPIC chromatograms of the sugar profile of KH. The retention times of the identified sugars were galactose, 8.4 min; glucose, 9.384 min; sucrose, 10.309 min; fructose, 12.250 min; lactose, 20.667 min; and maltose, 29.784 min.

KH is a dark amber with low viscosity and has a sweet aroma with a mildly acidic taste. The physicochemical analysis (Table 3) and sugar profile (Table 4) of KH were found to comply with the Malaysian KH Standard [3]. Figure 2 shows the HPIC chromatogram of the KH sugar profile. Meanwhile, the metal and mineral analyses reported here are in the range of other studies, as shown in Table 5.

### 3.2. GC-MS Semivolatile Organic Compound Analysis

**Figure 3:**
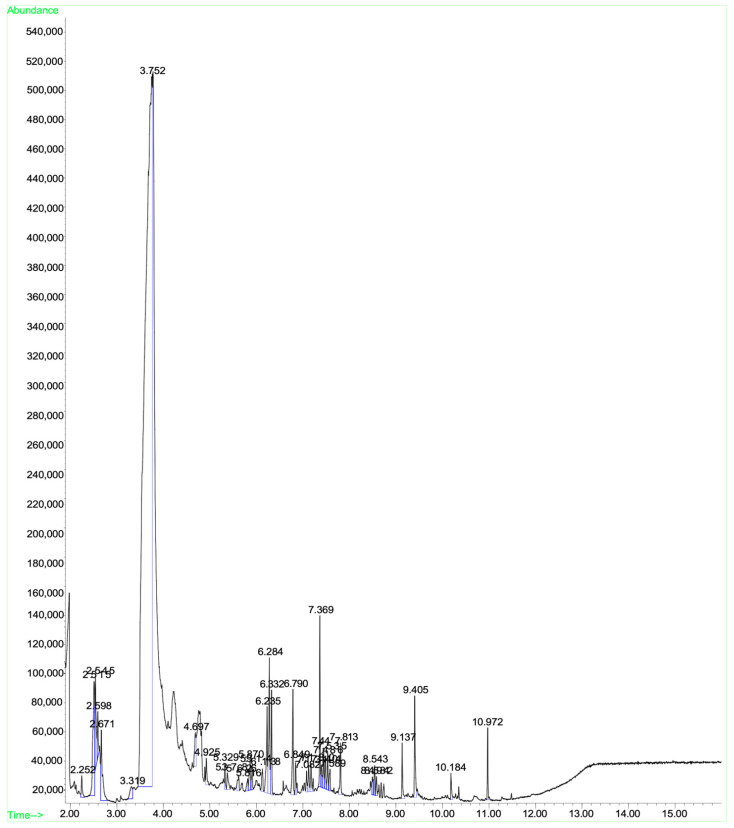
GC-MS spectrum of the semivolatile organic compounds present in KH.

A mass spectral analysis of KH identified six semivolatile compounds (Table 6) including 2,4-Dimethylhept-1-ene-(retention time (RT): 2.515, 2.598, 2.671 min), Tetradecane-(RT: 6.790 min), 2,4-Di-tert-butylphenol (RT: 7.369 min), n-Hexadecanoic acid-(RT: 9.405 min) and Octadecanoic acid (RT: 10.184 min), and z-10-Octdecen-1-ol acetate-(RT: 10.972 min). All compounds were identified by comparing their mass spectra with those found in the NIST mass spectral library. Figure 3 shows the GC-MS spectrum of the semivolatile organic compounds present in KH.

### 3.3. Effects of KH on Oestrus Cycle and Body Weight Gain

**Figure 4:**
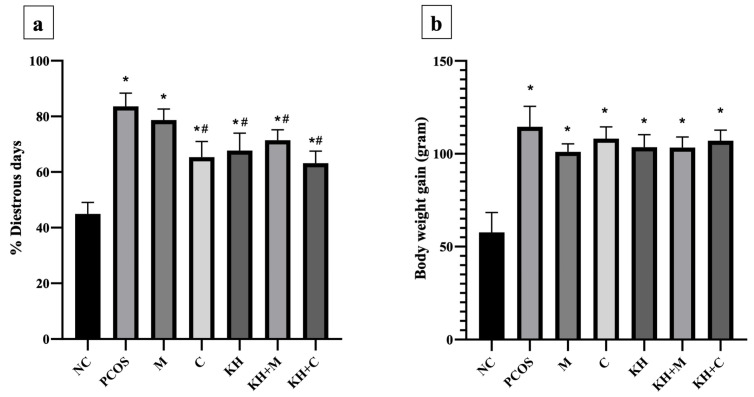
Effects of KH on (**a**) the percentage of dioestrus days and (**b**) rat body weight gain. NC: normal control; PCOS: Untreated PCOS; M: PCOS + Metformin; C: PCOS + Clomiphene; KH: PCOS + Kelulut honey; KH + M: PCOS + Kelulut honey + Metformin; KH + C: PCOS + Kelulut honey + Clomiphene. * *p* < 0.05 significance against the normal control group, ^#^ *p* < 0.05 significance against the untreated PCOS group. *n* = 6 per treatment group.

The percentage of dioestrus days (Figure 4a) was the lowest in the normal control group (44.94 ± 1.68%). Letrozole induction caused dioestrus days to increase significantly (*p* < 0.05) in untreated PCOS rats compared with normal control rats (83.59 ± 1.95% vs. 44.94 ± 1.68%, *p* < 0.05). Treatment with clomiphene (65.37 ± 2.29%), KH only (67.75 ± 2.55%), KH with metformin (71.42 ± 1.53%), and KH with clomiphene (63.18 ± 1.78%) significantly reduced the percentage of dioestrus days (*p* < 0.05) compared with the untreated PCOS group (83.59 ± 1.95%). Meanwhile, no significant difference in the percentage of dioestrus days was recorded among the clomiphene group, KH group, combined KH + metformin group, and combined KH + clomiphene group. Additionally, treatment with metformin alone (78.67 ± 1.62%) did not change the percentage of dioestrus days compared with the untreated PCOS group. 

Meanwhile, Figure 4b illustrates the effects of KH on body weight gain. Letrozole induction significantly increased the body weight gain in all PCOS rats compared with the normal control group. However, no significant difference was recorded between the untreated PCOS rats and any other treatment groups.

### 3.4. Effect of KH on Fasting Blood Glucose and Insulin Levels

**Figure 5:**
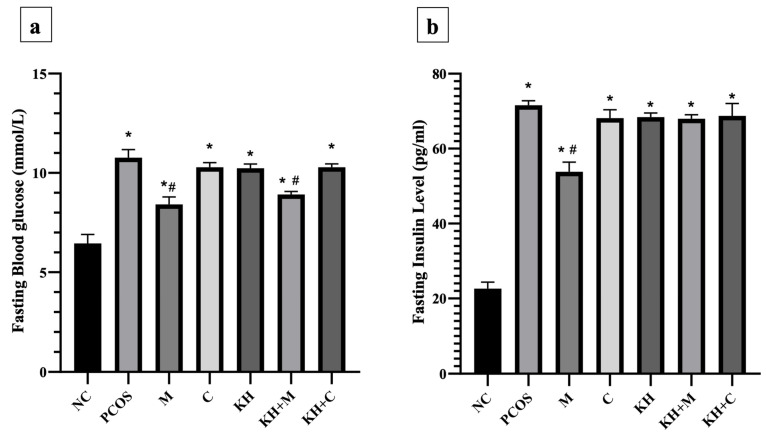
Effects of KH on the (**a**) fasting blood glucose and (**b**) insulin levels. NC: normal control; PCOS: Untreated PCOS; M: PCOS + Metformin; C: PCOS + Clomiphene; KH: PCOS + Kelulut honey; KH + M: PCOS + Kelulut honey + Metformin; KH + C: PCOS + Kelulut honey + Clomiphene. * *p* < 0.05 significance against the normal control group, ^#^ *p* < 0.05 significance against the untreated PCOS group. *n* = 6 per treatment group.

The effect of KH on fasting blood glucose levels is shown in Figure 5a. Blood glucose levels were significantly elevated (*p* < 0.05) in all PCOS rats compared with normal control rats (6.45 ± 0.18 mmol/L). The untreated PCOS group had the highest blood glucose (10.77 ± 0.17 mmol/L). Treatment with metformin or combined KH + metformin significantly reduced the fasting blood glucose compared with the untreated PCOS rats (8.42 ± 0.15 mmol/L, 8.92 ± 0.06 mmol/L vs. 10.77 ± 0.17 mmol/L, *p* < 0.05). We found that treatment with KH (10.23 ± 0.08 mmol/L), clomiphene (10.28 ± 0.09 mmol/L), or combined KH + clomiphene (10.28 ± 0.07 mmol/L) did not result in a significant reduction in blood glucose levels compared with the untreated PCOS rats (10.77 ± 0.17 mmol/L). 

Figure 5b illustrates the effects of KH on insulin levels. Insulin levels were significantly increased in all PCOS rats compared with normal control rats (*p* < 0.05). Treatment with metformin significantly reduced the insulin level compared with the untreated PCOS rats (53.83 ± 1.04 pg/mL vs. 71.60 ± 0.49 pg/mL, *p* < 0.05). Treatment with clomiphene (68.14 ± 0.92 pg/mL), KH (68.41 ± 0.45 pg/mL), combined KH + metformin (67.99 ± 0.43 pg/mL), or combined KH + clomiphene (68.75 ± 1.36 pg/mL) did not reduce the insulin levels compared with the untreated PCOS rats (71.60 ± 0.49 pg/mL). 

### 3.5. Effect of KH on Serum Testosterone, Oestradiol, Progesterone, LH, and FSH

**Figure 6:**
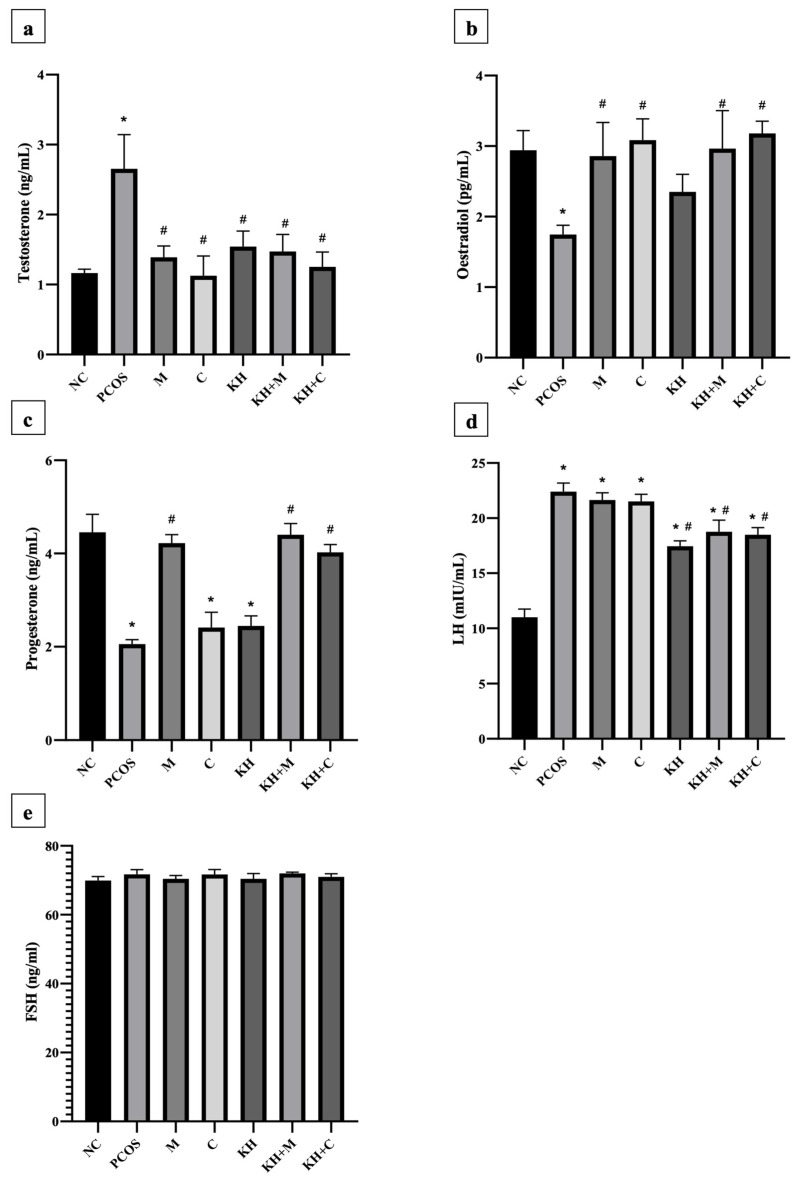
Effects of KH on serum (**a**) testosterone, (**b**) oestradiol, (**c**) progesterone, (**d**) LH, and (**e**) FSH levels. NC: normal control; PCOS: Untreated PCOS; M: PCOS + Metformin; C: PCOS + Clomiphene; KH: PCOS + Kelulut honey; KH + M: PCOS + Kelulut honey + Metformin; KH + C: PCOS + Kelulut honey + Clomiphene. * *p* < 0.05 significance against the normal control group, ^#^ *p* < 0.05 significance against the untreated PCOS group. *n* = 6 per treatment group.

Figure 6a demonstrates the effect of KH on testosterone levels. Testosterone levels were significantly elevated in untreated PCOS rats as compared with normal control rats (2.65 ± 0.19 ng/mL vs. 1.16 ± 0.02 ng/mL, *p* < 0.05). The elevated testosterone level was significantly reversed (*p* < 0.05) by treating the rats with clomiphene (1.12 ± 0.12 ng/mL), combined KH + clomiphene (1.25 ± 0.08 ng/mL), metformin (1.38 ± 0.07 ng/mL), combined KH + metformin (1.47 ± 0.09 ng/mL), and KH (1.54 ± 0.09 ng/mL). However, no significant differences were recorded among the treatment groups. Meanwhile, as shown in Figure 6b, oestradiol levels were significantly reduced in the untreated PCOS rats compared with the normal control group (1.74 ± 0.05 pg/mL vs. 2.94 ± 0.11 pg/mL, *p* < 0.05). Treatment with metformin (2.86 ± 0.19 pg/mL), combined KH + metformin (2.96 ± 0.22 pg/mL), clomiphene (3.08 ± 0.12 pg/mL), or combined KH + clomiphene (3.18 ± 0.07 pg/mL) significantly increased the oestradiol levels (*p* < 0.05) as compared with the untreated PCOS rats (1.74 ± 0.05 pg/mL). On the other hand, treatment with KH alone did not increase the oestradiol levels compared with the untreated PCOS rats.

As shown in Figure 6c, progesterone levels were significantly reduced in untreated PCOS rats compared with the normal control group (2.05 ± 0.04 ng/mL vs. 4.45 ± 0.15 ng/mL, *p* < 0.05). Only treatment with KH + metformin (4.40 ± 0.09 ng/mL), metformin (4.22 ± 0.07 ng/mL), and combined KH + clomiphene (4.02 ± 0.06 ng/mL) significantly increased the progesterone levels (*p* < 0.05) compared with the untreated PCOS rats (2.05 ± 0.04 ng/mL). Treatment with clomiphene (2.41 ± 0.13 ng/mL) and KH alone (2.44 ± 0.08 ng/mL) did not increase the progesterone levels compared with the untreated PCOS rats (2.05 ± 0.04 ng/mL). Meanwhile, Figure 6d illustrates that letrozole induction caused LH levels to increase significantly in untreated PCOS rats compared with the normal control rats (22.40 ± 0.32 mIU/mL vs. 11.01 ± 0.3 mIU/mL, *p* < 0.05). Treatment with KH alone (17.45 ± 0.19 mIU/mL), combined KH + clomiphene (18.48 ± 0.27 mIU/mL), and combined KH + metformin (18.75 ± 0.43 mIU/mL) significantly reduced the levels of LH compared with untreated PCOS rats (*p* < 0.05). No reduction in LH levels was recorded when the PCOS rats were treated with metformin (21.64 ± 0.27 mIU/mL) or clomiphene alone (21.52 ± 0.26 mIU/mL). However, no changes in FSH levels were found in any groups (Figure 6e). 

### 3.6. Effect of KH on Ovarian Oxidative Stress

**Figure 7:**
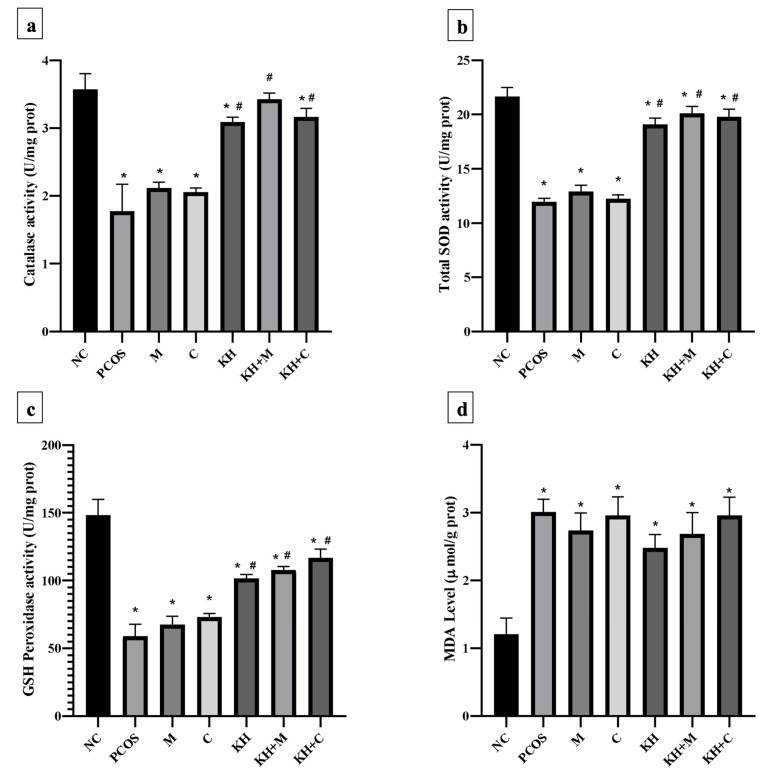
Effects of KH on ovarian (**a**) catalase activity, (**b**) total SOD activity, (**c**) GSH peroxidase activity, and (**d**) MDA levels. NC: normal control; PCOS: Untreated PCOS; M: PCOS + Metformin; C: PCOS + Clomiphene; KH: PCOS + Kelulut honey; KH + M: PCOS + Kelulut honey + Metformin; KH + C: PCOS + Kelulut honey + Clomiphene. * *p* < 0.05 significance against the normal control group, ^#^ *p* < 0.05 significance against the untreated PCOS group. *n* = 6 per treatment group.

Figure 7a illustrates the effect of KH on catalase activity in ovarian tissues. Letrozole induction decreased the catalase activity in untreated PCOS rats compared with the normal control group (1.78 ± 0.16 U/mg prot vs. 3.57 ± 0.09 U/mg prot, *p* < 0.05). This reduction was significantly reversed (*p* < 0.05) by treatment with KH + metformin (3.43 ± 0.04 U/mg prot), KH + Clomiphene (3.17 ± 0.05 U/mg prot), and KH alone (3.09 ± 0.03 U/mg prot), whereas treatment with metformin (2.12 ± 0.03 U/mg prot) or clomiphene alone (2.06 ± 0.03 U/mg prot) did not increase the catalase activity compared with the untreated PCOS rats. 

Total SOD activity in the ovarian tissues is shown in Figure 7b. SOD activity was found to be decreased in untreated PCOS rats as compared with the normal control group (11.97 ± 0.12 U/mg prot vs. 21.68 ± 0.33 U/mg prot, *p* < 0.05). Treatment with KH (19.11 ± 0.23 U/mg prot), combined KH + metformin (20.12 ± 0.26 U/mg prot), and combined KH + clomiphene (19.79 ± 0.28 U/mg prot) significantly increased the total SOD activities (*p* < 0.05) compared with the untreated PCOS rats (11.97 ± 0.12 U/mg prot). However, treatment with metformin (12.90 ± 0.23 U/mg prot) or clomiphene alone (12.24 ± 0.14 U/mg prot) did not increase the total SOD activity compared with the untreated PCOS rats.

Figure 7c demonstrates the effect of different treatments on GSH peroxidase activity in ovarian tissues. GSH peroxidase level was reduced in the untreated PCOS rats compared with the normal control rats (59.09 ± 4.29 U/mg prot vs. 148.22 ± 5.88 U/mg prot, *p* < 0.05). As in SOD and catalase analyses, treatment with KH (101.72 ± 1.39 U/mg prot), combined KH + clomiphene (116.66 ± 3.27 U/mg prot), and combined KH + metformin (107.76 ± 1.26 U/mg prot) significantly increased the GSH peroxidase activity (*p* < 0.05) as compared with the untreated PCOS rats (148.22 ± 5.88 U/mg prot). Treatment with metformin (67.33 ± 3.19 U/mg prot) or clomiphene alone (73.14 ± 1.33 U/mg prot) did not increase the GSH peroxidase activity compared with the untreated PCOS group. 

MDA level in the ovarian tissues (Figure 7d) was found to be significantly increased in untreated PCOS rats (3.01 ± 0.09 µmol/g prot) and all the other treatment groups compared with the normal control rats (1.21 ± 0.12 µmol/g prot). However, no differences in MDA levels were recorded in any treatment groups. 

## 4. Discussion

The physicochemical analysis of honey is essential for evaluating its quality and nutritional content. In this study, the physicochemical findings of KH complied with the range provided by the Malaysian Standard for stingless bee honey [3]. Our sample’s moisture content was lower than other KH analyses [40,41,42], which may suggest a better shelf life. It has been noted that the environmental conditions during harvesting and storage have an impact on the moisture content of KH. Honey with a high water content has a greater potential for fermentation, making its preservation and storage more challenging [43]. In addition, the value of HMF is a commonly accepted indicator of honey freshness. In a freshly obtained honey samples, HMF is typically absent and increases over time. HMF is a by-product of the breakdown of simple carbohydrates, particularly fructose. The HMF content has been reported to be affected by a number of variables, including heating, storage conditions, honey pH, and honey adulterated with sugars [44]. We recorded a very low value of HMF (<0.1 mg/100 g), which implies a good quality of the honey sample.

This study also revealed that KH has lower glucose and fructose values than other analyses of KH [39,42]. However, a review by Nordin et al. that analysed stingless bee honey physicochemical characteristics around the globe found that the range of glucose and fructose values was between 12.5 g/100 g and 75.7 g/100 g, with which our KH sample complies [2]. KH contains a lower sugar value than Apis sp bee honey [2]. The Malaysian Standard for stingless bee honey has determined a value of fructose and glucose (sum) to be not more than 85.0 g/100 g [3]. On the other hand, the mineral content of the honey is reported to relate to its nutritional benefit and depends on its botanical and geographical origin [39,45]. The mineral elements we reported were within the permissible values set by the World Health Organization (WHO) [46,47]. In minimal amounts, some heavy metals are nutritionally essential for human health. In contrast, a high concentration of heavy metals leads to toxicity which has the potential to cause disease in humans. The poisoning is due to these heavy metals’ inability to be metabolised by the body, thus leading to the heavy metals being accumulated to toxic levels within the human tissues without being degraded. Cobalt, molybdenum, and nickel are heavy metals of no biological importance and were not detected in our sample [48].

We demonstrated the semivolatile organic compounds identified in KH, which revealed six main chemical compounds, namely, 2,4-Dimethylhept-1-ene, Tetradecane, 2,4-Di-tert-butylphenol, n-Hexadecanoic acid, Octadecanoic acid, and z-10-Octadecen-1-ol acetate. The compound 2,4-Dimethylhept-1-ene is reported to play a role in human metabolites [49]. In contrast, tetradecane is noted as a phytochemical found in *Prosopis farcta* plants [50] and almonds [51]. Furthermore, a study reported that 2,4-Di-tert-butyl phenol is an antifungal and antioxidant bioactive compound purified from a newly isolated *Lactococcus* sp. [52]. In comparison, n-Hexadecanoic acid was reported to have anti-inflammatory [53] and cytotoxic properties [54]. Interestingly, octadecanoic acid or its synonym, stearic acid, was found to inhibit tumour development in a rat mammary carcinoma model induced by nitroso-methyl urea (NMU) [55]. Furthermore, a study comparing stearic acid with other saturated fatty acids in human studies has confirmed that stearic acid possesses cholesterol-lowering properties [56,57,58]. Another study showed that stearic acid (19 g/day) taken by healthy males could enhance their thrombogenic and atherogenic risk factor profiles [59]. Thus, the various health properties of semivolatile organic compounds found in KH enhanced its nutritional and antioxidative value.

As demonstrated in our previous study [28], KH did not affect the blood glucose levels in PCOS rats. This agrees with previous observations which found that daily intake of KH for 30 days caused no changes in fasting blood glucose in patients who have impaired fasting blood glucose [60]. Furthermore, we demonstrated that metformin treatment or combining KH with metformin reduced fasting blood glucose. This was expected due to metformin’s validated fasting glucose-lowering effect [61]. In this study, clomiphene demonstrated no effect on fasting blood glucose and insulin levels. Meanwhile, another study revealed that clomiphene does not improve glucose tolerance tests in PCOS rats [34]. In contrast, Emam et al. found an improvement in the fasting glucose levels of PCOS rats with clomiphene treatment [62]. A study reported that clomiphene increases insulin-like growth factor binding protein-1 and reduces insulin-like growth factor-I but does not correct insulin resistance associated with women with polycystic ovarian syndrome [63].

The oestrus cycle is an expression of the female reproductive system since it reflects the status of the ovary, uterus, and hormonal physiology. We demonstrated that KH treatment could normalise the oestrus cycle in PCOS rats, and this effect is comparable to clomiphene. Furthermore, the combination of KH with metformin and clomiphene showed significant normalisation of the oestrus cycle. These results strengthen our previous finding which revealed oestrus cycle improvement in PCOS-induced rats treated with KH [28]. Previously, Tualang honey has been demonstrated to improve the bisphenol A-induced disruption of the oestrus cycle [64]. Hence, KH may be explored more for its potential to enhance the female menstrual cycle.

According to Sohaei et al., women with PCOS are overweight or obese in 38–88% of cases [65]. Clinically significant improvements in PCOS symptoms are seen with a modest weight loss of 5–10% [66]. In accordance with previous findings [28], our current study showed that KH treatment for 35 days did not affect the rat body weight gain. Previously, a study showed that six-week KH supplementation to high-fat diet-induced obese rat models could reduce the rat body weight and BMI [8]. The difference in the duration of honey treatment may contribute to the disparity of the findings. According to Atangwho et al., the duration of honey supplementation determines the body weight alteration [67]. Therefore, longer treatment duration may be needed to explore the KH effect on body weight.

Disturbance in sex steroid hormones was among the main findings in women with PCOS and PCOS animal models [68,69]. As validated in numerous animal PCOS induction studies [68], we demonstrated increased testosterone and LH levels but decreased progesterone and oestradiol levels with PCOS induction. However, we could not find any changes in the FSH level. With KH treatment, the testosterone level was significantly reversed to near normal. A similar trend was recorded in the clomiphene and metformin groups or in the combination of KH with both drugs. In agreement with previous studies [62,70,71], metformin and clomiphene were reported to reverse the elevated testosterone level in letrozole-induced PCOS rats. In women with PCOS, metformin causes a rapid decrease in LH-stimulated testosterone secretion [72]. Meanwhile, another study reported that clomiphene treatment does not affect testosterone levels in PCOS-induced rats [34]. Clomiphene possesses oestrogenic and anti-oestrogenic properties, but its exact mode of action is yet to be known. Clomiphene stimulates the release of the gonadotropins, FSH and LH, which leads to the development and maturation of the ovarian follicle, ovulation, and subsequent development and function of the corpus luteum, thus resulting in pregnancy [73].

As for the LH levels, KH significantly reversed the increment induced by PCOS induction. Interestingly, a combination of KH with clomiphene and metformin reduced LH levels but not in clomiphene or metformin-only groups. This suggests a synergistic effect of KH with metformin and clomiphene. Similarly, Ndeingang et al. also found that clomiphene treatment does not affect the LH levels in PCOS rats [34]. Meanwhile, Ibrahim et al. discovered that metformin treatment reduced LH increment in PCOS-induced rats [71]. In a previous study, six months of metformin treatment successfully reduced the LH levels in women with PCOS [74]. The improvement in testosterone and LH levels in this study could be explained by our previous finding in which KH ameliorated the altered cystic follicles, antral follicles, and corpus luteum in PCOS-induced rat [28]. This improvement of the folliculogenesis process by KH can hinder the follicular hyperandrogenism and restore the sex steroid-related mechanism alteration in PCOS. In addition, a study proposed that local Nigerian honey may regulate the pituitary gland by modulating the feedback mechanism to alter the sex steroid hormonal level [75]. Meanwhile, another study also demonstrated honey could alter the hypothalamic-pituitary-adrenal axis [76].

In this study, we demonstrated that KH did not affect oestradiol, progesterone, or FSH levels. Serum oestradiol and FSH levels in letrozole-induced PCOS rats showed variations in different studies, as revealed by Ryu et al. [68]. Furthermore, a study that used the same model of letrozole-induced PCOS rats found that treatment with letrozole, metformin, and clomiphene did not affect the FSH levels [34]. According to a report, honey could be oestrogenic or anti-oestrogenic depending on its concentration [32,77,78]. In another study, female rats supplemented with Tualang honey for eight weeks demonstrated no changes in their oestradiol levels [79]. Meanwhile, other researchers have proved that Tualang honey reduces oestradiol and progesterone levels in ovariectomised rats [80]. However, Ismail et al. found no difference in testosterone, progesterone, and oestradiol levels with Gelam honey supplementation in ovariectomised rats [32].This may suggest further study on the effect of honey on the sex steroid hormone. In this study, metformin and clomiphene treatment caused oestradiol levels to increase, as similar findings have recorded previously [34,81]. Interestingly, while KH alone did not affect the oestradiol and progesterone levels in this study, the combination of KH with metformin or clomiphene increased both hormones significantly. This again suggests that KH has a synergistic effect with metformin and clomiphene.

Supplementation with KH in PCOS-induced rats significantly improved their oxidative stress status. We found that KH significantly increased catalase, total SOD, and GSH peroxidase activities compared with the untreated PCOS rats. In fact, higher values of catalase, total SOD, and GSH peroxidase activities were recorded when combining KH with clomiphene or metformin, in which treatment with the drugs alone did not improve the oxidative stress status in PCOS rats. Meanwhile, we found that the MDA level was not affected by KH. Our findings strengthen the findings regarding the antioxidative properties of KH, which are recorded in two other studies demonstrating that KH increases SOD and GSH levels in testicular oxidative damage [10] and increases SOD in an osteoporosis rat model [11]. Another study recorded findings similar to ours, in which letrozole induction did not alter the MDA level in rat ovaries [34]. The KH antioxidant effect is attributed to its phenolic content as suggested in previous studies [82,83]. The positive results shown by the KH and combination of KH with metformin or clomiphene in improving catalase, SOD, and GSH peroxidase activities were concomitant with its effect on improving testosterone and LH levels. This implies an interplay between the reduction of oxidative stress and the enhancement of physiological hormonal processes manifested by the oestrus cycle improvement seen in these groups. 

Reactive oxygen species and oxidative stress markers such as SOD, glutathione peroxidase, and catalase have been reported to have a regulatory role in both physiological and pathological processes of the oestrous cycle, folliculogenesis, oocyte maturation, ovarian steroidogenesis, and sex steroid hormone level [84,85,86]. Furthermore, as in our PCOS-induced rats, GSH peroxidase activity was found to be significantly reduced in the follicular fluid of tobacco-smoking women. In addition, GSH peroxidase activities were higher in fertilised oocytes than in non-fertilised oocytes [87]. In another study, a mitochondrial malfunction with reduced GSH levels and O_2_ consumption was found in PCOS patients with insulin resistance [88]. Park et al. proved that the inhibition of catalase causes DNA damage and chromosome misalignment during meiotic maturation in mouse oocytes [89]. Meanwhile, in vitro studies demonstrated that LH could increase the mRNA and protein levels of SOD and catalase in the bovine corpus luteum [90]. This may explain the normalisation of the oestrus cycle, testosterone, and LH level with improvement in SOD, glutathione peroxidase, and catalase seen with KH treatment on PCOS rats.

## 5. Conclusions

Taken together, our results show that KH and its combination with metformin or clomiphene improve oxidative stress, hormonal profile, and oestrus cycle in rats with PCOS. These results may provide a basis for future studies to discover the potential use of KH as a complementary treatment for women with PCOS.

## Figures and Tables

**Table 1:** The ICP-OES operating conditions.

Nebulizer	Micromist
RF generator (W)	1550
Argon flow rate (L min^−1^)	0.85
Nebuliser pump (rps)	0.10
Scanning condition	Number of replicates 3, dwelling time 1 s
H2 flow (L min^−1^)	3.5
He flows (L min^−1^)	4.0

**Table 2:** GC/MS parameters for the determination of SVOCs in KH.

GC Parameters	
Inlet mode	Splitless
Splitless time (min)	16
Carrier gas, flow, flow rate	Helium, constant pressure 10 psi, 1.7 mL/min
Oven	50 °C, 0.1 min
Chromatographic column	30 m × 0.25 mm internal diameter × 0.5 µm film thickness DB-UI-8270D ULTRA INERT (Agilent Technologies, Santa Clara, CA, USA)
MS Parameters	
Transfer line temperature (°C)	300
Source temperature (°C)	230
Ionisation mode	Electron ionisation (EI)
Electron energy (eV)	70
Acquisition mode	Full scan 40–650 m/z
MS Library	NIST MS Search 2.2 (Gaithersburg, MD, USA)

**Table 3:** Comparison of the physicochemical analysis of our KH and Malaysian KH Standard (Raw).

Analysis	KH	Malaysian KH Standard (Raw) [3]
Moisture Content	14.5 g/100 g	Not more than 35.0%
Ash Content	0.1 g/100 g	1.0 g/100 g
Hydroxyl Methyl Fulfural	<0.1 mg/100 g	Not more than 30.0 mg/kg
Free Acidity	269 meq/kg	2.5 to 3.8 pH
Diastase Number (DN)	<3 Schade Unit	Not stated

**Table 4:** Comparison of sugar contents in KH and Malaysian Standard KH (Raw).

Sugar Analysis	Value	Malaysian Standard KH (Raw) [3]
Fructose	9.6 g/100 g	Fructose and glucose (sum), not more than 85.0 g/100 g
Glucose	7.9 g/100 g	Fructose and glucose (sum), not more than 85.0 g/100 g
Sucrose	<0.100 g/100 g	Not more than 7.5 g/100 g
Maltose	0.845 g/100 g	Not more than 9.5 g/100 g
Lactose	<0.100 g/100 g	Not stated
Galactose	<0.100 g/100 g	Not stated
Total sugars	18.3 g/100 g	Not stated

**Table 5:** Comparison of metal and mineral contents in our KH and other analyses.

Metal and Mineral Analysis	Result (mg/kg)	Other Studies (mg/kg)
Calcium	361	59.513–191.9 [12,39]
Antimony	<0.100	No report found
Iron	0.9	6.57–10.90 [12,39]
Arsenic	<0.100	0.019 [39]
Potassium	894	370.65–732.2 [12,39]
Magnesium	40.7	10.09–33.81 [12,39]
Cadmium	<0.100	0.002–0.03 [12,39]
Sodium	36.4	108.78–589.7 [12,39]
Phosphorus	15.4	0.206 [39]
Sulphur	32.8	No report found
Lead	<0.100	0.154 [39]
Tin	2.89	No report found
Mercury	<0.050	0.022 [39]

**Table 6:** Semivolatile organic compounds identified in KH.

Retention Time (min)	GC-MS Semivolatile Organic Compounds	Molecular Weight (Da)	Cas. No.
2.515	2,4-Dimethylhept-1-ene	126.24	19549-87-2
2.598	2,4-Dimethylhept-1-ene	126.24	19549-87-2
2.671	2,4-Dimethylhept-1-ene	126.24	19549-87-2
6.790	Tetradecane	198.39	629-59-4
7.369	2,4-Di-tert-butylphenol	206.32	96-76-4
9.405	n-Hexadecanoic acid	256.42	57-10-3
10.184	Octadecanoic acid	284.5	57-11-4
10.972	z-10-Octadecen-1-ol acetate	310.5	Not found

## Data Availability

The data is contained within the article.
